# Diet-related knowledge, attitudes, and behaviors among young and middle-aged individuals with high-normal blood pressure: A cross-sectional study in China

**DOI:** 10.3389/fpubh.2022.898457

**Published:** 2022-09-02

**Authors:** Tingyu Mu, Rixiang Xu, Qianyin Zhu, Lingshan Chen, Die Dong, Jiayi Xu, Cuizhen Shen

**Affiliations:** ^1^School of Nursing, Zhejiang Chinese Medical University, Hangzhou, China; ^2^School of Nursing, Anhui University of Chinese Medicine, Hefei, China; ^3^School of Humanities and Management, Zhejiang Chinese Medical University, Hangzhou, China

**Keywords:** high-normal blood pressure, diet, knowledge, attitudes, behaviors, influencing factors, China

## Abstract

**Background:**

Dietary modifications play an important role in the prevention and management of high-normal blood pressure (BP). The aim of this study was to investigate diet-related knowledge, attitudes, and behaviors, and the socio-demographic determinants of these, among young and middle-aged Chinese individuals with high-normal BP.

**Methods:**

Data from the 2015 China Health and Nutrition Survey (CHNS) were analyzed in this study. A total of 1,756 subjects with high-normal BP were included. A chi-square test and binary logistic regression analysis were conducted to identify the risk factors toward diet-related knowledge, attitudes, and behaviors.

**Results:**

A total of 37.4% of the participants knew about the Chinese Food Pagoda (CFP) or the Dietary Guidelines for Chinese Residents (DGCR). Overall, 39.8% of the subjects were classified as having adequate diet-related knowledge literacy, 27.8% reported positive diet-related attitudes to healthy eating, and 35.3% reportedly looked for nutrition knowledge. Of note, 72.4% and 80.1% of the participants reported liking to eat fruits and vegetables, respectively. Individuals with a middle school education [odds ratio (OR) = 1.784, 95% CI = 1.236–2.576], high school/vocational education (OR = 1.944, 95% CI = 1.305–2.896), and college degree or above (OR = 2.089, 95% CI = 1.341–3.322), who were living in a rural area (OR = 1.311, 95% CI = 1.048–1.639), proactively looking for nutrition knowledge (OR = 1.529, 95% CI = 1.227–1.906), and reported liking to eat vegetables (OR = 1.939, 95% CI = 1.409–2.688), were more likely to have sufficient dietary knowledge literacy. Managers (OR = 1.655, 95% CI = 1.039–2.635) were more likely to have positive dietary attitudes. Female gender (OR = 1.396, 95% CI = 1.089–1.790), high school/vocational school education (OR = 2.071, 95% CI = 1.269–3.379), college degree and above (OR = 2.207, 95% CI = 1.262–3.862), knowledge about the CFP or DGCR (OR = 8.138, 95% CI = 6.326–10.468), and sufficient dietary knowledge literacy (OR = 1.338, 95% CI = 1.050–1.705) were associated with an increased likelihood of looking for nutrition knowledge.

**Conclusion:**

Individuals with high-normal BP, predominantly males, living in rural area, with lower education, farmers, workers, service workers, and workers in the non-government employment unit may have poor diet-related knowledge, attitudes, and behaviors.

## Introduction

Hypertension is a common chronic disease and a major risk factor for cardiovascular diseases (1). Effective blood pressure (BP) management is key to reducing the morbidity and mortality of coronary heart disease and stroke (2). High-normal BP is an intermediate state between normal BP and hypertension. A better understanding of the population with high-normal BP and a focus on this population for hypertension prevention will help address the increasing prevalence of hypertension (3). The current definition of high-normal BP differs between countries and regions. In 2003, the seventh report of the Joint National Committee on Prevention, Detection, Evaluation, and Treatment of High Blood Pressure (JNC7) (4) defined high-normal BP as systolic BP (SBP) of 120–139 mmHg and/or diastolic BP (DBP) of 80–89 mmHg. Furthermore, SBP between 120 and 129 mmHg and DBP under 80 mmHg are considered to be elevated BP according to the American College of Cardiology/American Heart Association (ACC/AHA) 2017 guidelines (5). According to the International Society of Hypertension Global Hypertension Practice Guidelines (2020), SBP between 130 and 139 mmHg and/or DBP between 85 and 89 mmHg is considered high-normal BP (6). The 2018 Chinese guidelines for the management of hypertension define high-normal BP as SBP between 120 and 139 mmHg and/or DBP between 80 and 89 mmHg (7). This study adopts this definition of high-normal BP, given that the sample for this study is obtained from the Chinese population.

As a result of increasingly unhealthy lifestyles, the number of people with high-normal BP has continued to grow (8). The prevalence of high-normal BP in some areas is even higher than the prevalence of hypertension in the general population (9). Globally, the prevalence of high-normal BP in healthy adults is reported to be 36.3% (10). In developing countries, the prevalence is reported to range from 30.7 to 47.3% (11–15). According to data reported by Xu T (16), the prevalence of high-normal BP in China is 36.4%, with the average age of patients reported to be 44.0 ± 16.0 years. High-normal BP is a known risk factor for cardiovascular disease and an independent risk factor for hypertension. A 15-year follow-up study found that, compared to individuals with BP under 120/80 mmHg, those with high-normal BP had a 78% increased risk of cardiovascular disease, a 77% increased risk of coronary heart disease, and a 79% increased risk of stroke, and the risk of cardiovascular death was also increased by 1.50 times (17). Therefore, it is of great importance to manage individuals with high-normal BP in order to reduce the prevalence of hypertension.

Dietary modifications play an important role in the prevention and management of high-normal BP. According to the AHA guidelines, individuals with high-normal BP should undertake dietary modifications (e.g., reducing sodium intake and increasing the consumption of fresh fruits, vegetables, and low-fat dairy products) (18, 19). The theory of knowledge, attitudes/beliefs, and behavior (KAB) suggests that individual health behavior consists of three consecutive processes, namely, acquiring knowledge, generating beliefs, and forming behaviors (20). Dietary knowledge is the basis of individual diet-related behavior change (20). However, hypertension-related knowledge and behaviors are lower in the high-normal BP population in Wuxing District, Huzhou City, China (a total of 297 participants with high-normal BP are investigated) (21), and <50% of people in Shanghai with a risk of hypertension maintain a healthy diet (22). In addition, knowledge, attitudes, and behaviors are influenced by socio-demographic factors (e.g., gender, age, educational level, and income) (23).

Therefore, this study was based on a national nutrition survey [2015 China Health and Nutrition Survey (CHNS)] to examine the diet-related knowledge, attitudes, and behaviors of the Chinese individuals with high-normal BP. The findings can inform interventions to improve the diet-related knowledge, attitudes, and behaviors of individuals with high-normal BP, which may help to delay or mitigate the onset of hypertension.

## Methods

### Study design and sample

The data for this study were obtained from a nationally representative cohort survey (CHNS), which is an international collaborative project between the Carolina Population Center at the University of North Carolina at Chapel Hill and the National Institute for Nutrition and Health (NINH, formerly the National Institute of Nutrition and Food Safety) at the Chinese Center for Disease Control and Prevention (CCDC). This survey was designed to examine the effects of health, nutrition, and family planning policies and programmes implemented by national and local governments and to examine how the social and economic transformation of the Chinese society is affecting the health and nutritional status of its population. CHNS used a multistage random-cluster sampling process to select samples from 15 provinces in China, including Liaoning, Shandong, Jiangsu, Henan, Hunan, Hubei, Guangxi, Guizhou, Heilongjiang, Zhejiang, Yunnan, and Shanxi, and three municipalities (Beijing, Chongqing, and Shanghai). The survey was updated every 2–4 years, with a total of 10 waves currently available (1989, 1991, 1993, 1997, 2000, 2004, 2006, 2009, 2011, and 2015). This study used data from the latest wave (2015). As of August 2018, a total of 220 community samples, 7,200 household samples, and 30,000 resident samples were included.

**Figure 1:**
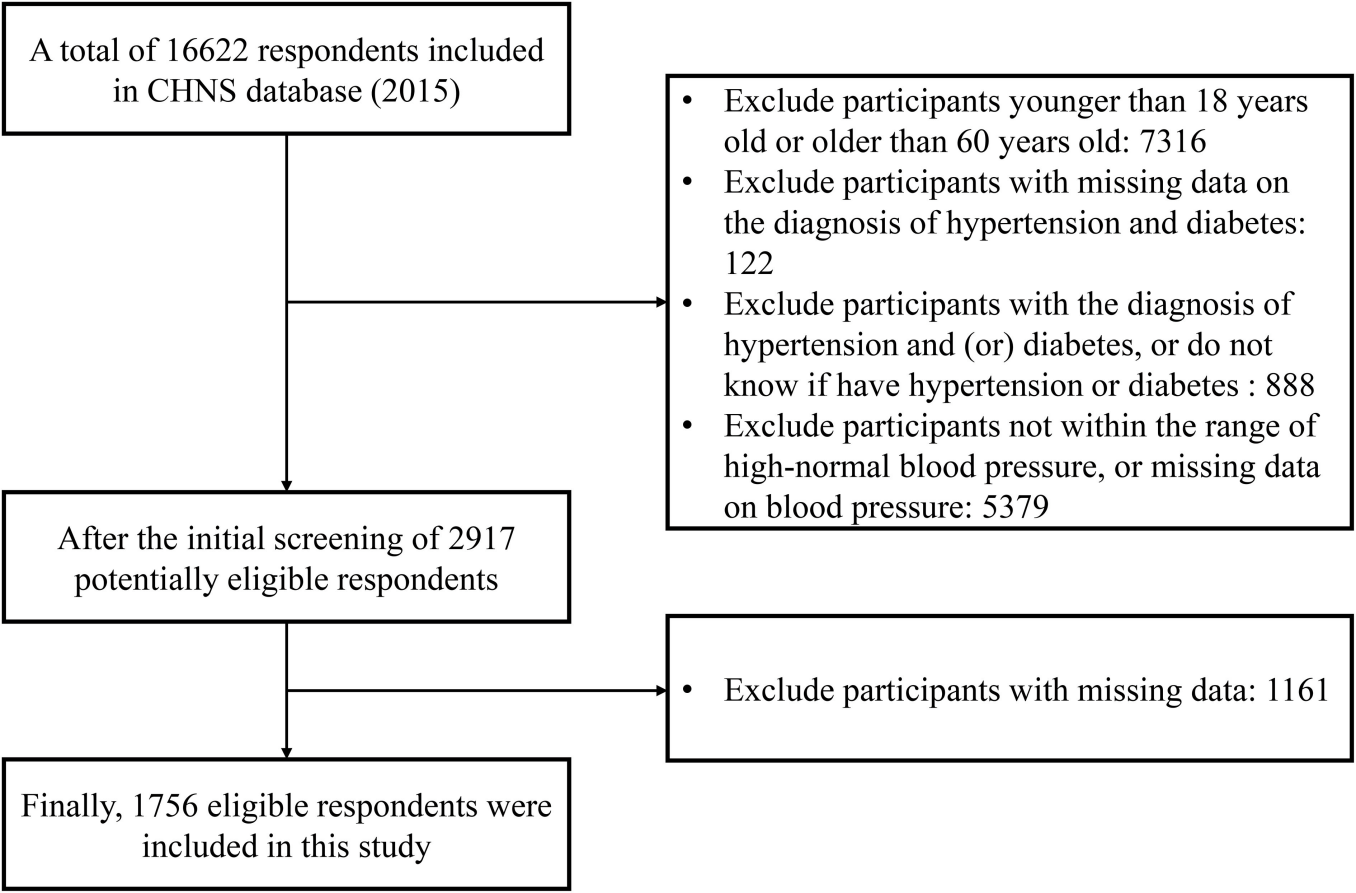
Flowchart of subject recruitment and eligibility.

The original dataset included data from 16,622 respondents. Respondents were eligible for inclusion in this study if they were aged ≥18 years or <60 years, had SBP between 120 and 139 mmHg and/or DBP between 80 and 89 mmHg (7) and were not diagnosed with hypertension or diabetes. Respondents were excluded if they had missing data with the diagnosis of hypertension and diabetes, BP, and diet-related variables. After the application of the inclusion and exclusion criteria, a total of 1,756 eligible respondents were included in this study. The screening process to determine eligibility is shown in Figure 1. All data used in this study were obtained from the official CHNS website (https://www.cpc.unc.edu/projects/china). Therefore, the author's institution did not require Institutional Review Board approval.

### Variables

#### Diet-related knowledge

Two indicators were used to assess diet-related knowledge. The first indicator was knowledge of the Chinese Food Pagoda (CFP) or Dietary Guidelines for Chinese Residents (DGCR). The question in the survey asked: “Do you know about the Chinese Food Pagoda or the Dietary Guidelines for Chinese Residents?” (yes/no). The second indicator was having sufficient dietary knowledge literacy. The second indicator had 17 dietary questions, including 10 positive questions (Q1, Q3, Q5, Q7, Q8, Q9, Q10, Q11, Q13, and Q17) and seven negative questions (Q2, Q4, Q6, Q12, Q14, Q15, and Q16). The response options for the 17 dietary knowledge questions were “strongly disagree,” “disagree,” “neutral,” “agree,” “strongly agree,” and “don't know.” These variables were transformed into dichotomous variables. For the 10 positive questions, responses of “strongly agree” or “agree” were given 1 point; otherwise, a score of 0 was given. For the seven negative questions, responses of “strongly disagree” or “disagree” were given 1 point; otherwise, a score of 0 was given. The scores to these 17 questions were summed and a total score ≥14 was considered to reflect adequate dietary knowledge literacy (24). Cronbach's alpha for the 17 dietary questions was 0.834 in this study.

#### Diet-related attitudes

The indicator for diet-related attitudes was based on participants' views on the importance of healthy eating. The question in the survey was: “How important is eating a healthy diet in your life?” and the response options were “not important at all,” “not very important,” “neutral,” “very important,” “the most important” and “don't know.” Responses of “the most important” or “very important” were taken to represent a positive attitude (20).

#### Diet-related behaviors

Three indicators were selected to assess diet-related behaviors. The first indicator was related to nutrition knowledge. The question in the survey was “Do you proactively look for nutrition knowledge?” (yes/no). A response of “yes” was considered to reflect positive behavior. The second indicator and the third indicator were related to eating fruits and vegetables, respectively. The questions in the survey were “How much do you like fruits?” (“dislike very much,” “dislike,” “neutral,” “like,” “like very much,” and “don't eat”) and “How much do you like vegetables?” (“dislike very much,” “dislike,” “neutral,” “like,” “like very much,” and “don't eat”). Responses of “like” or “like very much” were taken to represent positive behavior (20).

#### Covariates

Various control variables were included in the analyses, including gender, age, location of residence, education level, marital status, primary occupation, and type of work unit for primary occupation. These covariates were included in the analyses based on previous studies or preliminary univariate analyses indicating that they are confounders of diet-related knowledge, attitudes, and behaviors. Age was divided into two categories (18–44 years and 45–59 years; 45 years was considered the threshold between young and middle age in China) (25). The location of residence was categorized as urban or rural. Education level was categorized as elementary school and below, middle school, high school/vocational school, or college degree and above. Marital status was categorized as never married, married, divorced, widowed, or separated.

Primary occupation was categorized as technical workers (e.g., senior professional/technical worker and junior professional/technical worker), managers (e.g., administrator/executive/manager), office staff (e.g., secretary/office helper), farmers (e.g., farmer/fisherman/hunter), skilled/unskilled worker, service workers (e.g., driver/housekeeper/cook/waiter), and others (e.g., army officer/policeman/athlete/actor). Type of work unit for primary occupation was categorized as government employed (e.g., government department/state service/institute/state-owned enterprise), non-government employed (e.g., small collective enterprise/large collective enterprise/private and individual enterprise/three-capital enterprise), family contract farming, or unknown.

### Statistical analyses

The original data from the CHNS database were exported in DTA format to Microsoft EXCEL 2016 for data screening. Statistical analysis was performed using SPSS 25.0 (SPSS Inc., Chicago, IL, US). Descriptive statistics, including means and standard deviations (SDs), frequencies, and percentages were generated. The chi-squared test was used to compare the baseline characteristics between the diet-related knowledge, attitudes, and behaviors groups. Multivariate analysis models were constructed using the Enter algorithm to assess the net effect of each potential factor. Binary logistic regression was used to examine the associations between diet-related knowledge, attitudes, and behaviors and socio-demographic characteristics, after adjusting for possible confounding factors. *P*-values of <0.05 were considered statistically significant.

## Results

### Sample characteristics

A total of 1,756 eligible respondents were included in this study, with a mean age of 42.83 ± 9.41 years. The mean SBP and DBP were 123.50 ± 7.38 mmHg and 80.29 ± 4.88 mmHg, respectively. The majority of participants were male (58.8%) and were married with a spouse present (90.5%). Furthermore, 59.3% lived in rural areas and 52.8% were currently in non-government employment. Further detail on the sample characteristics can be found in Table 1.

**Table 1:** Sample characteristics of the Chinese respondents with high-normal blood pressure.

**Characteristics**	**Total (%)**	**Diet-related knowledge**						**Diet-related attitudes**			**Diet-related behaviors**								
		**CFP/DGCR**			**Dietary knowledge literacy**			**Positive**	**Negative**	***P***	**Look for nutrition knowledge**			**Eating fruits**			**Eating vegetables**		
		**Know**	**Don't know**	***P***	**Adequate**	**Inadequate**	***P***				**Positive**	**Negative**	***P***	**Positive**	**Negative**	***P***	**Positive**	**Negative**	***P***
**Gender**				0.040			0.078			0.846			<0.001			<0.001			<0.001
Male	1,032 (58.8)	365	667		393	639		285	747		329	703		680	352		796	236	
Female	724 (41.2)	291	433		306	418		203	521		291	433		591	133		611	113	
**Age**				0.419			0.787			0.411			0.164			0.541			0.116
18–44	915 (52.1)	350	565		367	548		262	653		337	578		668	247		720	195	
45–59	841 (47.9)	306	535		332	509		226	615		283	558		603	238		687	154	
**Location of residence**				<0.001			0.707			0.877			<0.001			0.185			
Urban	714 (40.7)	358	356		288	426		197	517		319	395		529	185		564	150	0.324
Rural	1,042 (59.3)	298	744		411	631		291	751		301	741		742	300		843	199	
**Education level**				<0.001			<0.001			0.096			<0.001			0.266			0.963
Elementary school and below	229 (13.1)	27	202		54	175		59	170		35	194		156	73		183	46	
Middle school	590 (33.7)	133	457		222	368		147	443		136	454		421	169		477	113	
High school/vocational School	482 (27.5)	204	278		208	274		137	345		196	286		354	128		385	97	
College degree and above	452 (25.8)	292	160		214	238		143	309		251	201		338	114		360	92	
**Marital status**				0.806			0.795			0.496			0.733			0.378			0.003
Never Married	112 (6.4)	41	71		46	66		33	79		38	74		82	30		76	36	
Married	1,587 (90.5)	595	992		628	959		445	1,142		558	1,029		1,148	439		1,288	299	
Divorced	29 (1.7)	11	18		11	18		5	24		12	17		19	10		23	6	
Widowed	23 (1.3)	7	16		12	11		5	18		9	14		19	4		18	5	
Separated	3 (0.2)	2	1		1	2		0	3		2	1		1	2		1	2	
**Primary occupation**				<0.001			<0.001			0.004			<0.001			0.007			0.703
Technical worker	275 (15.8)	185	90		135	140		82	193		155	120		216	59		224	51	
Manager	115 (6.6)	70	45		54	61		46	69		67	48		76	39		92	23	
Office staff	193 (11.1)	111	82		89	104		67	126		99	94		142	51		147	46	
Famer	307 (17.6)	52	255		91	216		85	222		67	240		201	106		240	67	
Worker	382 (22.0)	93	289		128	254		90	292		89	293		271	111		309	73	
Service worker	369 (21.1)	112	257		151	218		94	275		111	258		280	89		302	67	
Others	105 (6.0)	30	75		48	57		24	81		30	75		77	28		85	20	
**Type of work unit for primary occupation**				<0.001			<0.001			0.145			<0.001			0.017			0.738
Government employed	535 (31.1)	322	213		254	281		166	369		269	266		398	137		434	101	
Non-government employed	908 (52.8)	280	628		351	557		238	670		282	626		664	244		728	180	
Family contract farming	237 (13.8)	39	198		68	169		71	166		49	188		151	86		184	53	
Unknown	39 (2.3)	7	32		17	22		8	31		9	30		28	11		31	8	

CFP, Chinese Food Pagoda; DGCR, Dietary Guidelines for Chinese Residents.

### Diet-related knowledge, attitudes, and behaviors

**Figure 2:**
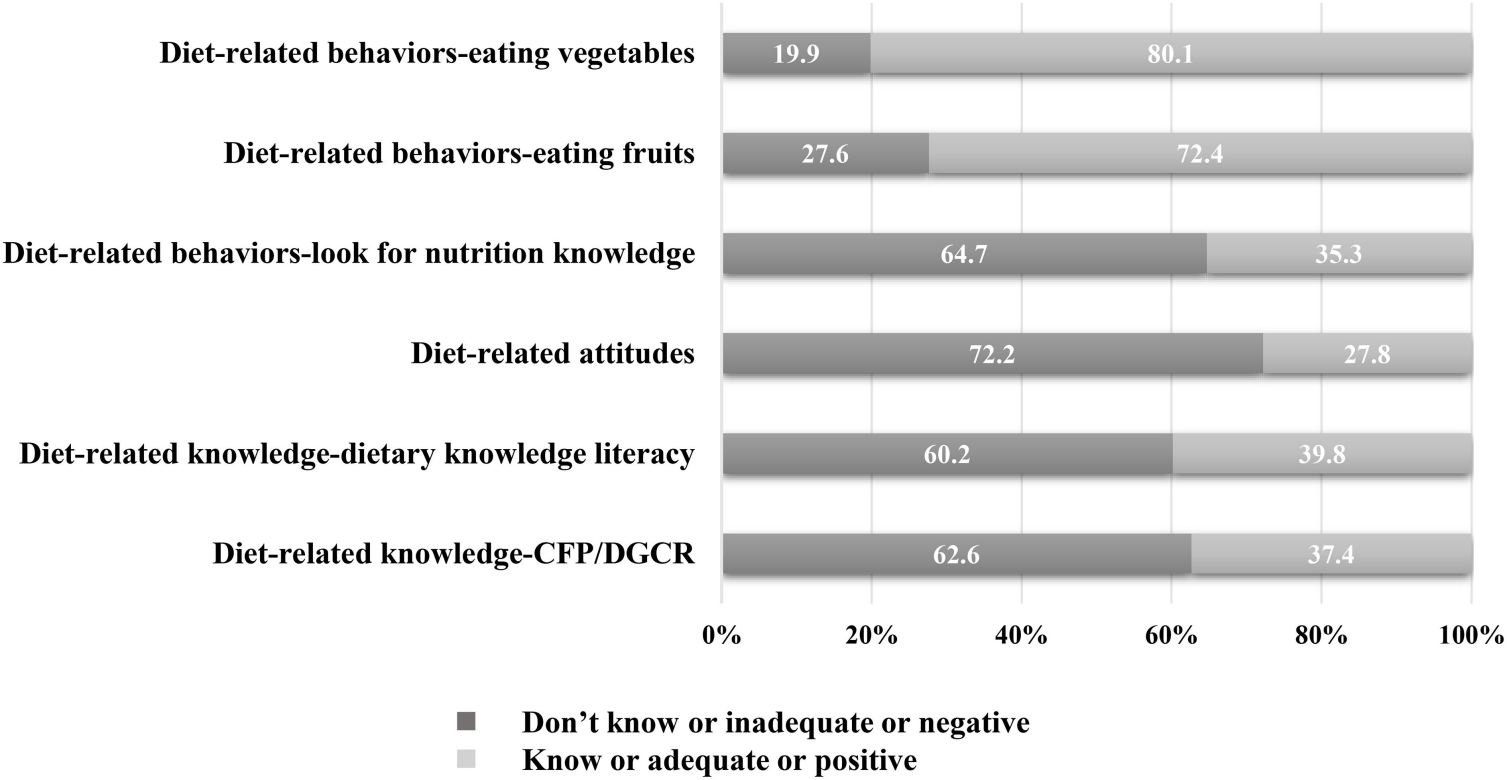
Percentage of diet-related knowledge, attitudes, and behaviors among participant with high-normal blood pressure. CFP, Chinese Food Pagoda; DGCR, the Dietary Guidelines for Chinese Residents.

**Figure 3:**
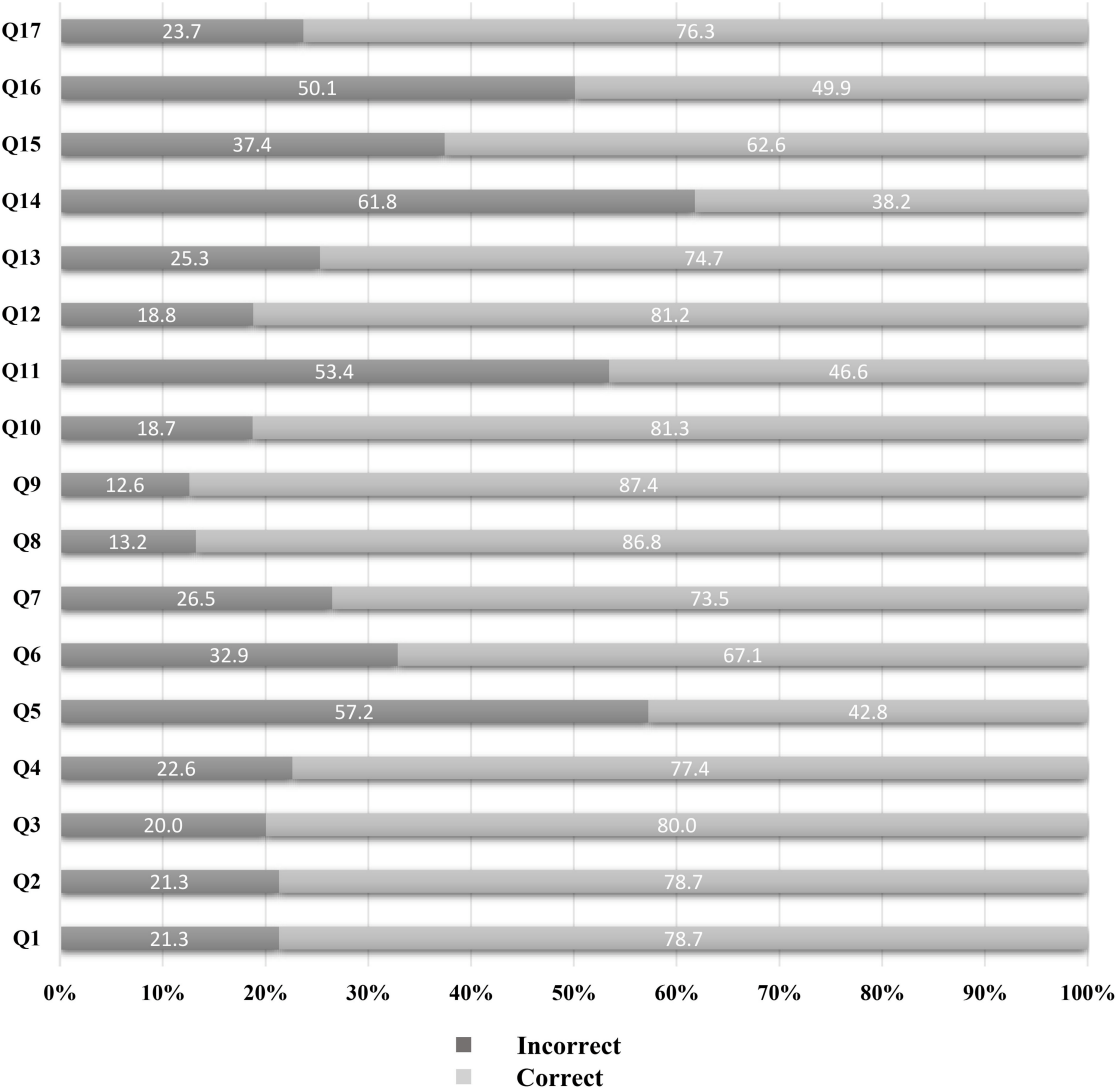
Proportion of answering on seventeen dietary knowledge questions among participant with high-normal blood pressure. Q1: Choosing a diet with a lot of fresh fruits and vegetables is good for one's health; Q2: Eating a lot of sugar is good for one's health; Q3: Eating a variety of foods is good for one's health; Q4: Choosing a diet high in fat is good for one's health; Q5: Choosing a diet with a lot of staple foods (rice and rice products and wheat and wheat products) is not good for one's health; Q6: Consuming a lot of animal products daily (fish, poultry, eggs, and lean meat) is good for one's health; Q7: Reducing the amount of fatty meat and animal fat in the diet is good for one's health; Q8: Consuming milk and dairy products is good for one's health; Q9: Consuming beans and bean products is good for one's health; Q10: Physical activities are good for one's health; Q11: Sweaty sports or other intense physical activities are not good for one's health; Q12: The heavier one's body is, the healthier he or she is; Q13: Eating salty foods can cause hypertension; Q14: Refined grains (rice and wheat flour) contain more vitamins and minerals than unrefined grains; Q15: Lard is healthier than vegetable oils; Q16: Vegetables contain more starch than staple foods (rice or wheat flour); Q17: Eggs and milk are the important sources of high-quality protein.

Figure 2 summarizes the results in relation to diet-related knowledge, attitudes, and behaviors. Overall, 37.4% of participants knew about CFP/DGCR, 39.8% had adequate dietary knowledge literacy, and 27.8% had positive diet-related attitudes. Furthermore, 35.3% of participants reported that they looked for nutrition knowledge, 72.4% liked eating fruits, and 80.1% liked eating vegetables. The detailed results for the 17 dietary knowledge questions are presented in Figure 3. The proportion of subjects who correctly answered each question ranged from 38.2 (Q14) to 87.4% (Q9). The questions with an error rate of >50% were Q14 (61.8%, r*efined grains (rice and wheat flour) contain more vitamins and minerals than unrefined grains*), Q5 (57.2%, *choosing a diet with a lot of staple foods (rice and rice products and wheat and wheat products*) is not good for one's health), Q11 (53.4%, *sweaty sports or other intense physical activities are not good for one's health*), and Q16 (50.1%, *vegetables contain more starch than staple foods (rice or wheat flour)*). Only 4.6% (*n* = 81) of participants gave correct answers to all 17 questions (Supplementary Table 1).

### Univariate analyses

The results of the univariate analyses are presented in Table 1. Gender (*P* = 0.040), location of residence (*P* < 0.001), education level (*P* < 0.001), primary occupation (*P* < 0.001), and type of work unit for primary occupation (*P* < 0.001) were associated with knowledge about CFP/DGCR. Dietary knowledge literacy was associated with education level (*P* < 0.001), primary occupation (*P* < 0.001), and type of work unit for primary occupation (*P* < 0.001). Diet-related attitudes were associated with primary occupation (*P* = 0.004). Gender (*P* < 0.001), location of residence (*P* < 0.001), education level (*P* < 0.001), primary occupation (*P* < 0.001), and type of work unit for primary occupation (*P* < 0.001) were associated with looking for nutrition knowledge. Gender (*P* < 0.001), primary occupation (*P* = 0.007), and type of work unit for primary occupation (*P* = 0.017) were associated with eating fruits. Gender (*P* < 0.001) and marital status (*P* = 0.003) were associated with eating vegetables.

### Multivariate analysis

The results of the binary logistic regression analyses examining the associations between the socio-demographic characteristics and diet-related knowledge, attitudes, and behaviors are shown in Table 2.

**Table 2:** Binary logistic regression analysis for the characteristics associated with diet-related knowledge, attitudes, and behaviors.

**Characteristics**	**Diet-related knowledge**				**Diet-related attitudes**		**Diet-related behaviors**					
	**CFP/DGCR**		**Dietary knowledge literacy**		**OR**	**95%CI**	**Looking for nutrition knowledge**		**Eating fruits**		**Eating vegetables**	
	**OR**	**95%CI**	**OR**	**95%CI**			**OR**	**95%CI**	**OR**	**95%CI**	**OR**	**95%CI**
**Gender (ref: male)**	1.04	0.80–1.35	1.10	0.89–1.36	0.98	0.78–1.24	1.40^**^	1.09–1.79	2.28^***^	1.79–2.89	1.66^***^	1.28–2.17
**Age (ref: 18–44)**	1.15	0.88–1.50	1.05	0.85–1.30	0.90	0.71–1.13	0.88	0.68–1.13	1.05	0.83–1.33	1.17	0.90–1.52
**Location of residence (ref: urban)**	0.72^*^	0.53–0.93	1.31^*^	1.05–1.64	1.11	0.88–1.42	0.87	0.67–1.13	0.93	0.73–1.19	1.20	0.92–1.57
**Education level (ref: Elementary school and below)**												
Middle school	1.79^*^	1.09–2.95	1.78^**^	1.24–2.58	0.97	0.67–1.41	1.28	0.81–2.04	1.12	0.78–1.60	1.06	0.70–1.59
High school/vocational school	2.62^***^	1.57–4.39	1.94^**^	1.31–2.90	1.09	0.72–1.63	2.07^**^	1.27–3.38	1.14	0.77–1.70	0.95	0.61–1.49
College degree and above	3.95^***^	2.21–7.07	2.09^**^	1.34–3.32	1.05	0.65–1.71	2.21^**^	1.26–3.86	1.08	0.67–1.76	0.96	0.56–1.64
**Marital status (ref: Never Married)**												
Married	1.35	0.82–2.25	0.90	0.59–1.38	0.98	0.63–1.52	1.27	0.77–2.10	0.88	0.55–1.40	1.84^**^	1.17–2.89
Divorced	1.08	0.37–3.20	0.87	0.35–2.13	0.54	0.19–1.58	1.90	0.69–5.25	0.77	0.30–1.96	1.68	0.61–4.69
Widowed	1.01	0.32–3.20	1.82	0.68–4.87	0.57	0.17–1.86	2.17	0.67–6.95	1.29	0.38–4.38	1.17	0.37–3.64
Separated	2.19	0.07–115.36	0.73	0.06–10.13	/	/	2.97	0.13–69.21	0.13	0.01–1.51	0.21	0.02–2.44
**Primary occupation (ref: technical worker)**												
Manager	0.72	0.42–1.25	0.97	0.61–1.53	1.66^*^	1.04–2.64	1.53	0.91–2.56	0.61	0.37–1.01	1.01	0.57–1.79
Office staff	0.72	0.46–1.12	0.93	0.64–1.37	1.28	0.86–1.91	1.02	0.66–1.56	0.72	0.46–1.13	0.78	0.49–1.24
Famer	0.33^**^	0.15–0.77	0.76	0.41–1.43	0.73	0.36–1.48	1.05	0.50–2.22	0.69	0.35–1.33	1.07	0.51–2.27
Worker	0.47^**^	0.30–0.75	0.73	0.49–1.08	0.82	0.53–1.25	0.66	0.42–1.04	0.86	0.55–1.34	1.34	0.82–2.19
Service worker	0.55^*^	0.35–0.87	0.93	0.63–1.36	0.88	0.58–1.33	0.81	0.52–1.26	0.96	0.61–1.51	1.20	0.74–1.96
Others	0.49^*^	0.27–0.89	1.09	0.66–1.81	0.67	0.37–1.19	0.74	0.41–1.36	0.99	0.55–1.77	1.35	0.70–2.59
**Type of work unit for primary occupation (ref: government employed)**												
Non-government employed	0.67^*^	0.49–0.92	0.87	0.66–1.13	0.99	0.74–1.32	1.11	0.81–1.52	1.00	0.74–1.36	0.87	0.62–1.22
Family contract farming	0.83	0.35–1.97	0.74	0.39–1.42	1.52	0.74–3.10	0.91	0.42–2.00	0.87	0.45–1.69	0.76	0.36–1.63
Unknown	0.32^*^	0.11–0.88	1.02	0.50–2.09	0.82	0.35–1.89	0.91	0.37–2.27	0.93	0.43–2.04	0.72	0.31–1.72
**Diet-related knowledge- CFP/DGCR(ref: don't know)**	/	/	/	/	1.3	0.99–1.72	8.14^***^	6.33–10.47	1.15	0.89–1.49	1.25	0.94–1.67
**Diet-related knowledge- dietary knowledge literacy (ref: negative)**	/	/	/	/	1.02	0.82–1.28	1.34^*^	1.05–1.71	1.66^***^	1.31–2.10	2.18^***^	1.66–2.87
**Diet-related attitudes (ref: negative)**	1.30	0.99–1.71	1.04	0.83–1.30	/	/	0.88	0.63–1.14	1.20	0.93–1.54	1.21	0.92–1.61
**Diet-related behaviors- looking for nutrition knowledge (ref: negative)**	8.31^***^	6.46–10.68	1.53^***^	1.23–1.91	0.88	0.67–1.15	/	/	/	/	/	/
**Diet-related behaviors- eating fruits (ref: negative)**	1.02	0.73–1.42	1.21	0.92–1.60	1.13	0.84–1.51	/	/	/	/	/	/
**Diet-related behaviors- eating vegetables (ref: negative)**	1.178	0.82–1.70	1.94^***^	1.41–2.67	1.14	0.82–1.58	/	/	/	/	/	/

* P < 0.05;

** P < 0.01;

*** P < 0.001.

CFP, Chinese Food Pagoda; DGCR, Dietary Guidelines for Chinese Residents.

#### Diet-related knowledge

##### Knowledge about the CFP or DGCR

Middle school education (*OR* = 1.794, *95% CI* = 1.092–2.949), high school/vocational school education (*OR* = 2.623, *95% CI* = 1.567–4.389), college degree and above (*OR* = 3.949, *95% CI* = 2.208–7.065), and proactively looking for nutrition knowledge (*OR* = 8.306, *95% CI* = 6.460–10.681) were related to an increased likelihood of being aware of the CFP or DGCR. Living in a rural area (*OR* =0.718, *95% CI* = 0.533–0.933), working as a famer (*OR* = 0.334, *95% CI* = 0.146–0.765), working as a skilled or unskilled worker (*OR* = 0.474, *95% CI* = 0.298–0.751), working as a service worker (*OR* = 0.552, *95% CI* = 0.352–0.867), and working in non-government employment (*OR* = 0.673, *95% CI* = 0.493–0.918) were associated with a reduced likelihood of being aware of the CFP or DGCR.

##### Having sufficient dietary knowledge literacy

Those with a middle school education (*OR* = 1.784, *95% CI* = 1.236–2.576), high school/vocational school education (*OR* = 1.944, *95% CI* = 1.305–2.896), college degree and above (*OR* = 2.089, *95% CI* = 1.341–3.322), and living in a rural area (*OR* = 1.311, *95% CI* = 1.048–1.639), who were proactively looking for nutrition knowledge (*OR* = 1.529, *95% CI* = 1.227–1.906) and liked eating vegetables (*OR* = 1.939, *95% CI* = 1.409–2.688) were more likely to have sufficient dietary knowledge literacy.

#### Diet-related attitudes

Those working as a manager (*OR* = 1.655, *95% CI* = 1.039–2.635) were more likely to have positive dietary attitudes.

#### Diet-related behaviors

##### Looking for nutrition knowledge

Female gender (*OR* = 1.396, *95% CI* = 1.089–1.790), high school/vocational school education (*OR* = 2.071, *95% CI* = 1.269–3.379), college degree and above (*OR* = 2.207, *95% CI* = 1.262–3.862), knowledge about the CFP or DGCR (*OR* = 8.138, *95% CI* = 6.326–10.468), and sufficient dietary knowledge literacy (*OR* = 1.338, *95% CI* = 1.050–1.705) were associated with an increased likelihood of looking for nutrition knowledge.

##### Eating fruits

Female gender (*OR* = 2.275, *95% CI* = 1.788–2.894) and sufficient dietary knowledge literacy (*OR* = 1.658, *95% CI* = 1.312–2.096) were associated with an increased likelihood of liking to eat fruits.

##### Eating vegetables

Female gender (*OR* = 1.664, *95% CI* = 1.275–2.171), married (*OR* = 1.836, *95% CI* = 1.169–2.885), and sufficient dietary knowledge literacy (*OR* = 2.181, *95% CI* = 1.659–2.871) were associated with an increased likelihood of liking to eat vegetables.

## Discussion

This study examined diet-related knowledge, attitudes, and behaviors among the Chinese individuals with high-normal BP. The findings indicated that relatively few individuals with high-normal BP knew about the CFP/DGCR (37.4%), had adequate dietary knowledge literacy (39.8%), had positive diet-related attitudes (27.8%), and looked for nutrition knowledge (35.3%). This can be compared with the Chinese adult residents (27.1% know about the CFP/DGCR, 34.3% have adequate dietary knowledge literacy, 24.3% hold positive attitudes toward healthy eating, and 27.6% reportedly look for nutrition knowledge) (20). Thus, in general, the diet-related knowledge, attitudes, and behaviors of individuals with high-normal BP were low. In this study, most individuals with high-normal BP reported liking to eat fruits (72.4%) and vegetables (80.1%); these percentages are much higher than the percentage of individuals who reported looking for nutrition knowledge. Thus, it is necessary to improve diet-related education and guidance for individuals with high-normal BP who are at a high risk of developing hypertension.

In relation to dietary knowledge literacy, the proportion of individuals who correctly answered all 17 questions was extremely low (4.6%). Furthermore, 61.8% of participants thought that refined grains (rice and wheat flour) contain more vitamins and minerals than unrefined grains, only 42.8% of participants thought that choosing a diet with a lot of staple foods (rice and rice products and wheat and wheat products) is not good for one's health, and 50.1% of participants believed that vegetables contain more starch than staple foods (rice or wheat flour). This indicates that many of these individuals with high-normal BP do not understand the nutritional contents of staple foods and vegetables. This misunderstanding could also affect their choice of suitable foods. Previous studies have shown that certain nutrients are associated with increased BP. Vitamin D, vitamin C, and B vitamins can impact BP (26–30). Reasonable intake of vitamins can have a lowering effect on BP, but insufficient intake may increase BP. Furthermore, increased intake of dietary fiber can also reduce BP (31). Whole grains provide more nutrients, such as B vitamins, minerals, dietary fiber, and healthy phytochemicals than refined grains (32). Moreover, increased intake of total cereal and vegetable fibers, but not fruit fiber, is associated with a decreased risk of hypertension in adults (33). Meanwhile, lycopene, anthocyanin, and resveratrol in vegetables and fruits have several cardiovascular beneficial effects such as antioxidative, anti-inflammatory, anti-atherogenic, and antiplatelet effects, improving endothelial function and BP control (34–37). Lycopene is a lipophilic unsaturated carotenoid found in red-colored fruits and vegetables such as tomatoes and watermelons. Lycopene has antihypertensive effects due to the inhibition of the angiotensin-converting enzyme (ACE) and block angiotensin II-induced vasoconstriction, oxidative stress, vascular smooth muscle cells phenotypic transformation, and production of inflammatory cytokines (36). Anthocyanins are water-soluble flavonoids commonly found in strawberries, blueberries, red grapes, and other berries. Anthocyanins have antihypertensive effects by inhibiting ACE activity and have also been shown to prevent hypertension (37). Resveratrol is a naturally occurring polyphenol found mostly in the skin of red grapes, peanuts, and several types of berries, which reduces BP by reducing sodium reabsorption and serum angiotensin II levels (34). However, in this study of the Chinese individuals with normal-high BP, many participants did not know the correct nutritional contents of refined grains, whole grains, and vegetables. Therefore, to improve the dietary management of people with high-normal BP, it is necessary to strengthen education related to the nutrients in refined grains, whole grains, and vegetables in order to improve their dietary knowledge.

The results of the regression analyses indicated that gender, location of residence, education level, marital status, primary occupation, and type of work unit for primary occupation had important influences on the diet-related knowledge, attitudes, and behaviors of individuals with high-normal BP. Diet-related knowledge and behaviors also influenced each other. In relation to diet-related knowledge, fewer individuals in rural areas knew about the CFP/DGCR, which is consistent with previous studies (20, 38). This may be related to the unbalanced urban–rural development in China; there may be less publicity of CFP/DGCR in rural areas. In addition, farmers, skilled/unskilled workers, service workers, and individuals in non-government employment were less aware of the CFP/DGCR. Therefore, the individual's primary occupation and the work unit should be taken into account when carrying out diet-related education. Compared with those in government employment, people with high-normal BP who were in non-government employment were less aware of the CFP/DGCR, which suggests that more attention should be paid to the dietary health and education of individuals in non-government employment. In relation to diet-related attitudes, managers with high-normal BP had better dietary attitudes than a technical worker. This may be related to education level. High-normal BP individuals with high education levels may have more channels through which to acquire dietary knowledge, thus resulting in more positive dietary attitudes. In relation to diet-related behaviors, women's diet-related behaviors were better than men's behaviors, which is consistent with previous studies (22). Women were more likely to be looking for dietary knowledge and eating fruits and vegetables than men in this study; the gender difference in fruit and vegetable intake has been widely confirmed (39). Research indicates that an increased intake of fruits and vegetables can effectively reduce the risk of cardiovascular disease (40). However, Chinese residents' actual intake of fruits and vegetables is not yet in line with international recommendations (41, 42), and some studies have even suggested a decline in the consumption of vegetables (43). Moreover, this study found that married people with high-normal BP were more likely to eat vegetables, which is a positive behavior. Therefore, diet-related behavioral interventions among the high-normal BP population are needed to promote increased consumption of fruits and vegetables and encourage the active acquisition of dietary knowledge. Moreover, attention should be paid to gender differences and the interaction between spouses in the process of diet-related behavioral interventions; peer support between husband and wife may promote a change in male dietary behaviors. Finally, this study found an interaction between dietary knowledge and dietary behaviors among people with high-normal BP, with sufficient dietary knowledge leading to positive dietary behaviors and vice versa.

People with high-normal BP are susceptible to hypertension. Based on KAB theory, the results of this study indicate that diet-related knowledge, attitudes, and behaviors among the high-normal BP population are at a low level. Therefore, it is necessary to change the diet-related knowledge, attitudes, and behaviors of this population by emphasizing the significance of an appropriate diet when imparting dietary knowledge.

This study has several limitations. First, the data for this study were obtained from the CHNS database. Given that the last wave of the CHNS was collected in 2015, these data may not fully reflect the current situation in China. Second, due to the limitations of the data structure and content of the CHNS database, only three indicators were selected (proactively looking for nutrition knowledge, eating fruits, and eating vegetables) as measures of diet-related behavior. These indicators may not fully reflect the diet-related behavior of the Chinese individuals with high-normal BP. Third, all indicators used in this study were obtained from individual self-reports and, therefore, may be subject to measurement error. Fourth, as there were substantial missing data for the economic income variable in the CHNS database, economic income was not included as a covariate in this study. Despite these limitations, this study systematically characterized the dietary status of the Chinese individuals with high-normal BP based on national data.

## Conclusion

The results of this study indicate that the levels of diet-related knowledge, attitudes, and behaviors among the Chinese individuals with high-normal BP are low. Therefore, diet-related knowledge, attitudes, and behaviors must be promoted among this group. Specifically, individuals with normal-high BP, predominantly males, living in a rural area, with lower education, and farmers, unskilled/skilled workers, service workers, and workers in non-government employment may have particularly poor diet-related knowledge, attitudes, and behaviors. Thus, health and dietary education and interventions must be directed at these groups. Such interventions would be beneficial for delaying or mitigating the onset of hypertension.

## Data availability statement

Publicly available datasets were analyzed in this study. This data can be found here: https://www.cpc.unc.edu/projects/china.

## Author contributions

TM designed the study, assessed the quality of the studies, and wrote the first draft of the manuscript. TM and RX conducted data analysis. CS monitored article quality and language polish. QZ, LC, DD, and JX extracted the data from CHNS according to the inclusion criteria and proofread these data. All authors contributed to and have approved the final manuscript.

## Funding

This study was funded by the Zhejiang Medical and Health Research Foundation (2022KY909).

## Conflict of interest

The authors declare that the research was conducted in the absence of any commercial or financial relationships that could be construed as a potential conflict of interest.

## Publisher's note

All claims expressed in this article are solely those of the authors and do not necessarily represent those of their affiliated organizations, or those of the publisher, the editors and the reviewers. Any product that may be evaluated in this article, or claim that may be made by its manufacturer, is not guaranteed or endorsed by the publisher.
