# Supplementary material for: Diet-related knowledge, attitudes, and behaviors among young and middle-aged individuals with high-normal blood pressure: A cross-sectional study in China
Source: Front Public Health. 2022 Sep 2;10:898457. doi: 10.3389/fpubh.2022.898457 (PMC9479537; doi:10.3389/fpubh.2022.898457)
Supplement: Supplementary file 1 [file Table_1.pdf]

Supplementally Table 1. Number and proportion of dietary knowledge literacy scores

Dietary Knowledge literacy Score	N(%)
0	28(1.6)
1	11(0.6)
2	19(1.1)
3	17(1.0)
4	40(2.3)
5	41(2.3)
6	47(2.7)
7	41(2.3)
8	64(3.6)
9	71(4.0)
10	116(6.6)
11	154(8.5)
12	184(10.5)
13	224(12.8)
14	208(11.8)
15	232(13.2)
16	178(10.1)
17	81(4.6)
